# Erratum for Aslam et al., “*Pseudomonas aeruginosa* ventricular assist device infections: findings from ineffective phage therapies in five cases”

**DOI:** 10.1128/aac.01800-24

**Published:** 2025-01-08

**Authors:** Saima Aslam, Dwayne Roach, Mikeljon P. Nikolich, Biswajit Biswas, Robert T. Schooley, Kimberly A. Bishop-Lilly, Gregory K. Rice, Regina Z. Cer, Theron Hamilton, Matthew Henry, Tiffany Luong, Ann-Charlott Salabarria, Laura Sisk-Hackworth, Andrey A. Filippov, Francois Lebreton, Lindsey Hall, Ran Nir-Paz, Hadil Onallah, Gilat Livni, Eran Shostak, Anat Wieder-Finesod, Dafna Yahav, Ortal Yerushalmy, Sivan Alkalay-Oren, Ron Braunstein, Leron Khalifa, Amit Rimon, Daniel Gelman, Ronen Hazan

## ERRATUM

Volume 68, no. 4, e01728-23, 2024, https://doi.org/10.1128/aac.01728-23. Kimberly A. Bishop-Lilly’s name should appear as shown in this erratum.

